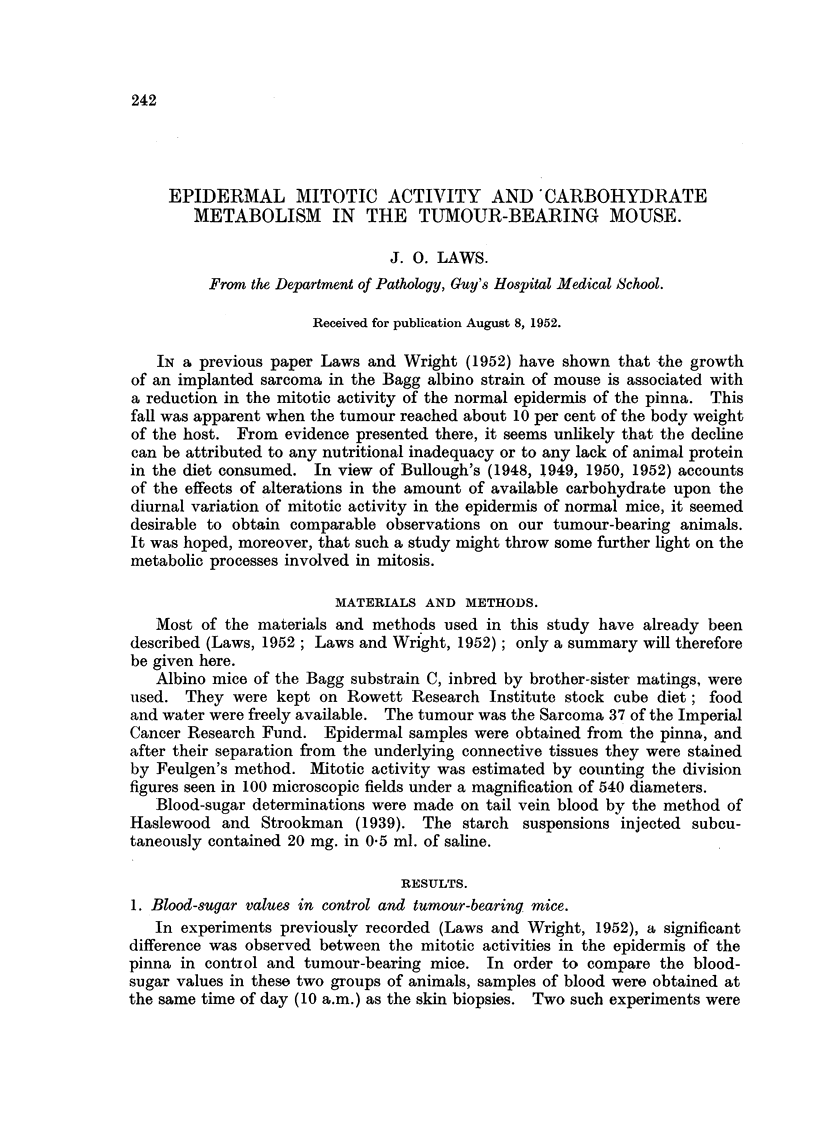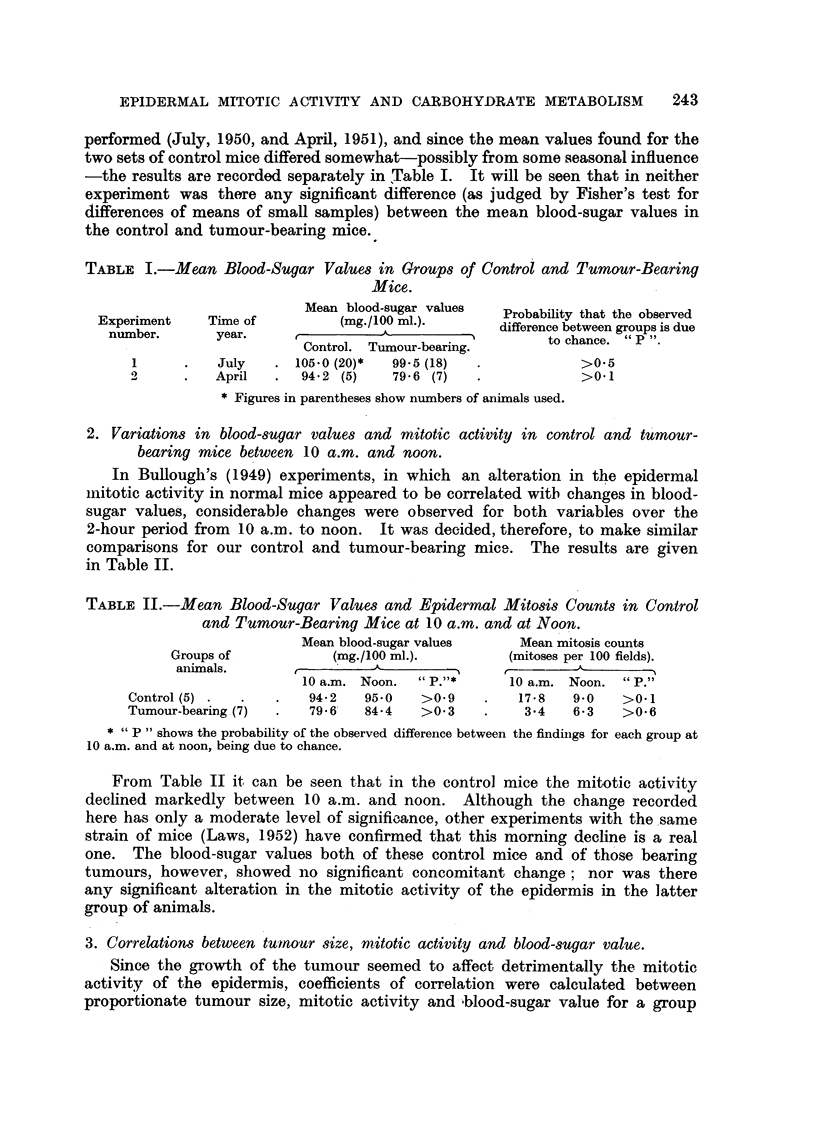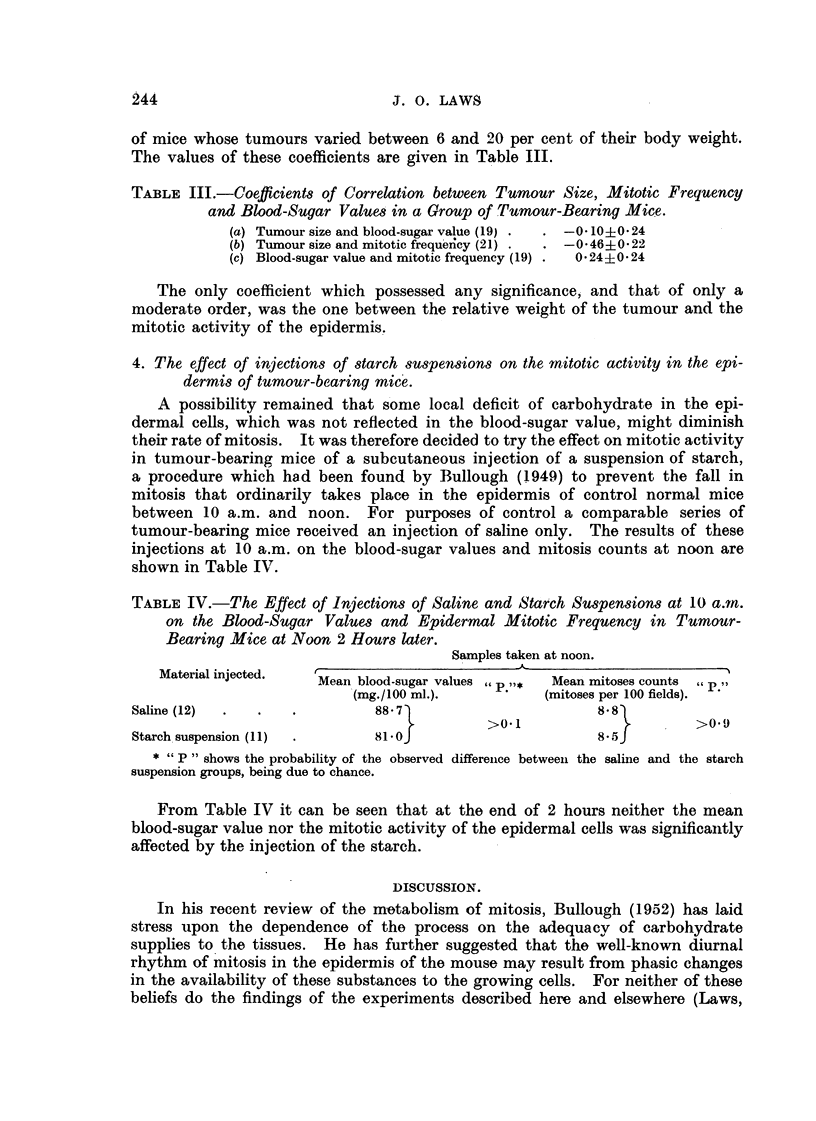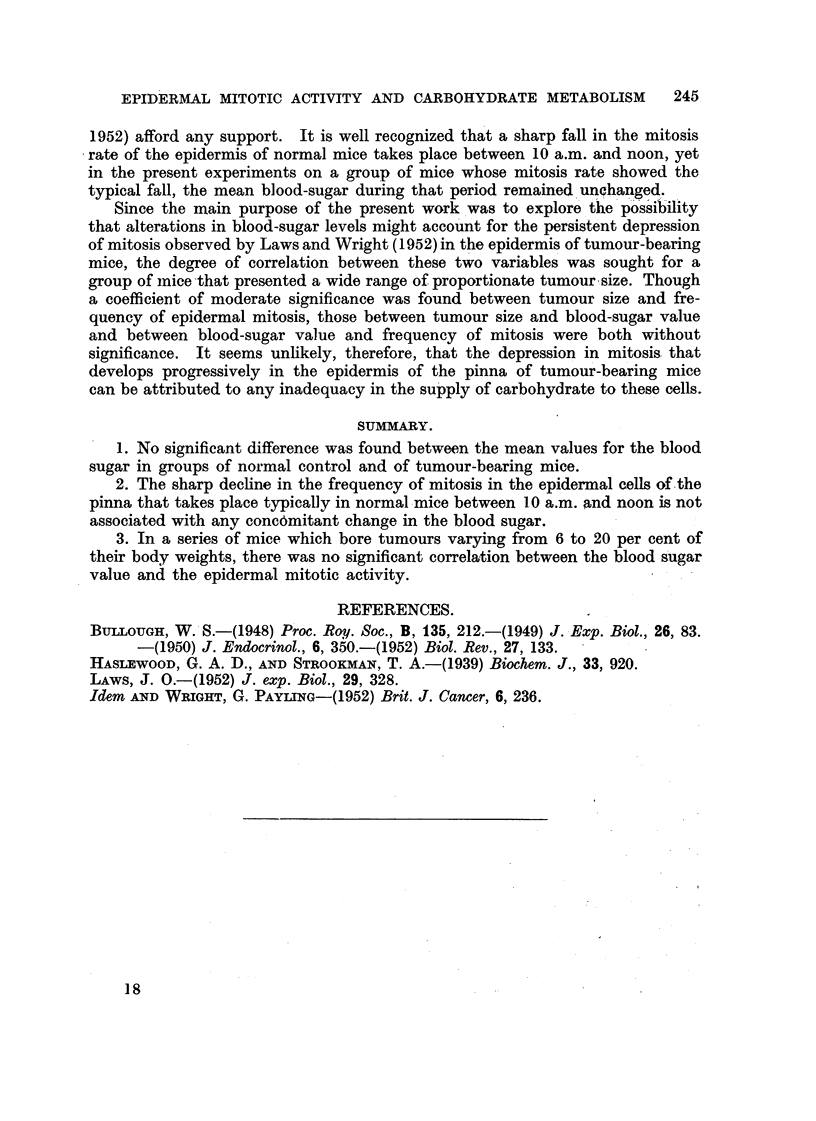# Epidermal Mitotic Activity and Carbohydrate Metabolism in the Tumour-bearing Mouse

**DOI:** 10.1038/bjc.1952.27

**Published:** 1952-09

**Authors:** J. O. Laws


### EPIDERMAL MITOTIC ACTIVITY AND CARBOHYDRATE METABOLISM IN THE TUMOUR-BEARING MOUSE.

J. O. LAWS.

*From the Department of Pathology, Guy's Hospital Medical School.*

Received for publication August 8, 1952.

IN a previous paper Laws and Wright (1952) have shown that the growth of an implanted sarcoma in the Bagg albino strain of mouse is associated with a reduction in the mitotic activity of the normal epidermis of the pinna. This fall was apparent when the tumour reached about 10 per cent of the body weight of the host. From evidence presented there, it seems unlikely that the decline can be attributed to any nutritional inadequacy or to any lack of animal protein in the diet consumed. In view of Bullough's (1948, 1949, 1950, 1952) accounts of the effects of alterations in the amount of available carbohydrate upon the diurnal variation of mitotic activity in the epidermis of normal mice, it seemed desirable to obtain comparable observations on our tumour-bearing animals. It was hoped, moreover, that such a study might throw some further light on the metabolic processes involved in mitosis.

### MATERIALS AND METHODS.

Most of the materials and methods used in this study have already been described (Laws, 1952 ; Laws and Wright, 1952) ; only a summary will therefore be given here.

Albino mice of the Bagg substrain C, inbred by brother-sister matings, were used. They were kept on Rowett Research Institute stock cube diet ; food and water were freely available. The tumour was the Sarcoma 37 of the Imperial Cancer Research Fund. Epidermal samples were obtained from the pinna, and after their separation from the underlying connective tissues they were stained by Feulgen's method. Mitotic activity was estimated by counting the division figures seen in 100 microscopic fields under a magnification of 540 diameters.

Blood-sugar determinations were made on tail vein blood by the method of Haslewood and Strookman (1939). The starch suspensions injected subcutaneously contained 20 mg. in 0·5 ml. of saline.

### RESULTS.

### 1. *Blood-sugar values in control and tumour-bearing mice.*

In experiments previously recorded (Laws and Wright, 1952), a significant difference was observed between the mitotic activities in the epidermis of the pinna in control and tumour-bearing mice. In order to compare the blood-sugar values in these two groups of animals, samples of blood were obtained at the same time of day (10 a.m.) as the skin biopsies. Two such experiments were performed (July, 1950, and April, 1951), and since the mean values found for the two sets of control mice differed somewhat—possibly from some seasonal influence —the results are recorded separately in Table I. It will be seen that in neither experiment was there any significant difference (as judged by Fisher's test for differences of means of small samples) between the mean blood-sugar values in the control and tumour-bearing mice.

TABLE I.—*Mean Blood-Sugar Values in Groups of Control and Tumour-Bearing Mice.*

| Experiment number. | Time of year. | Mean blood-sugar values (mg./100 ml.). Control. | Tumour-bearing. | Probability that the observed difference between groups is due to chance. "P". |
|---|---|---|---|---|
| 1 | July | 105·0 (20)* | 99·5 (18) | >0·5 |
| 2 | April | 94·2 (5) | 79·6 (7) | >0·1 |

* Figures in parentheses show numbers of animals used.

2. *Variations in blood-sugar values and mitotic activity in control and tumour-bearing mice between 10 a.m. and noon.*

In Bullough's (1949) experiments, in which an alteration in the epidermal mitotic activity in normal mice appeared to be correlated with changes in blood-sugar values, considerable changes were observed for both variables over the 2-hour period from 10 a.m. to noon. It was decided, therefore, to make similar comparisons for our control and tumour-bearing mice. The results are given in Table II.

TABLE II.—*Mean Blood-Sugar Values and Epidermal Mitosis Counts in Control and Tumour-Bearing Mice at 10 a.m. and at Noon.*

| Groups of animals. | Mean blood-sugar values (mg./100 ml.). | | | Mean mitosis counts (mitoses per 100 fields). | | |
|---|---|---|---|---|---|---|
| | 10 a.m. | Noon. | "P."* | 10 a.m. | Noon. | "P." |
| Control (5) | 94·2 | 95·0 | >0·9 | 17·8 | 9·0 | >0·1 |
| Tumour-bearing (7) | 79·6 | 84·4 | >0·3 | 3·4 | 6·3 | >0·6 |

* "P" shows the probability of the observed difference between the findings for each group at 10 a.m. and at noon, being due to chance.

From Table II it can be seen that in the control mice the mitotic activity declined markedly between 10 a.m. and noon. Although the change recorded here has only a moderate level of significance, other experiments with the same strain of mice (Laws, 1952) have confirmed that this morning decline is a real one. The blood-sugar values both of these control mice and of those bearing tumours, however, showed no significant concomitant change ; nor was there any significant alteration in the mitotic activity of the epidermis in the latter group of animals.

3. *Correlations between tumour size, mitotic activity and blood-sugar value.*

Since the growth of the tumour seemed to affect detrimentally the mitotic activity of the epidermis, coefficients of correlation were calculated between proportionate tumour size, mitotic activity and blood-sugar value for a group of mice whose tumours varied between 6 and 20 per cent of their body weight. The values of these coefficients are given in Table III.

TABLE III.—*Coefficients of Correlation between Tumour Size, Mitotic Frequency and Blood-Sugar Values in a Group of Tumour-Bearing Mice.*

| | | |
|---|---|---|
| (*a*) | Tumour size and blood-sugar value (19) . . | $-0\cdot10\pm0\cdot24$ |
| (*b*) | Tumour size and mitotic frequency (21) . . | $-0\cdot46\pm0\cdot22$ |
| (*c*) | Blood-sugar value and mitotic frequency (19) . | $0\cdot24\pm0\cdot24$ |

The only coefficient which possessed any significance, and that of only a moderate order, was the one between the relative weight of the tumour and the mitotic activity of the epidermis.

4. *The effect of injections of starch suspensions on the mitotic activity in the epidermis of tumour-bearing mice.*

A possibility remained that some local deficit of carbohydrate in the epidermal cells, which was not reflected in the blood-sugar value, might diminish their rate of mitosis. It was therefore decided to try the effect on mitotic activity in tumour-bearing mice of a subcutaneous injection of a suspension of starch, a procedure which had been found by Bullough (1949) to prevent the fall in mitosis that ordinarily takes place in the epidermis of control normal mice between 10 a.m. and noon. For purposes of control a comparable series of tumour-bearing mice received an injection of saline only. The results of these injections at 10 a.m. on the blood-sugar values and mitosis counts at noon are shown in Table IV.

TABLE IV.—*The Effect of Injections of Saline and Starch Suspensions at* 10 *a.m. on the Blood-Sugar Values and Epidermal Mitotic Frequency in Tumour-Bearing Mice at Noon* 2 *Hours later.*

| Material injected. | Samples taken at noon. | | | |
|---|---|---|---|---|
| | Mean blood-sugar values (mg./100 ml.). | " P."* | Mean mitoses counts (mitoses per 100 fields). | " P." |
| Saline (12) . . . | 88·7 | >0·1 | 8·8 | >0·9 |
| Starch suspension (11) . | 81·0 | | 8·5 | |

* " P " shows the probability of the observed difference between the saline and the starch suspension groups, being due to chance.

From Table IV it can be seen that at the end of 2 hours neither the mean blood-sugar value nor the mitotic activity of the epidermal cells was significantly affected by the injection of the starch.

### DISCUSSION.

In his recent review of the metabolism of mitosis, Bullough (1952) has laid stress upon the dependence of the process on the adequacy of carbohydrate supplies to the tissues. He has further suggested that the well-known diurnal rhythm of mitosis in the epidermis of the mouse may result from phasic changes in the availability of these substances to the growing cells. For neither of these beliefs do the findings of the experiments described here and elsewhere (Laws,

1952) afford any support. It is well recognized that a sharp fall in the mitosis rate of the epidermis of normal mice takes place between 10 a.m. and noon, yet in the present experiments on a group of mice whose mitosis rate showed the typical fall, the mean blood-sugar during that period remained unchanged.

Since the main purpose of the present work was to explore the possibility that alterations in blood-sugar levels might account for the persistent depression of mitosis observed by Laws and Wright (1952) in the epidermis of tumour-bearing mice, the degree of correlation between these two variables was sought for a group of mice that presented a wide range of proportionate tumour size. Though a coefficient of moderate significance was found between tumour size and frequency of epidermal mitosis, those between tumour size and blood-sugar value and between blood-sugar value and frequency of mitosis were both without significance. It seems unlikely, therefore, that the depression in mitosis that develops progressively in the epidermis of the pinna of tumour-bearing mice can be attributed to any inadequacy in the supply of carbohydrate to these cells.

### SUMMARY.

1. No significant difference was found between the mean values for the blood sugar in groups of normal control and of tumour-bearing mice.

2. The sharp decline in the frequency of mitosis in the epidermal cells of the pinna that takes place typically in normal mice between 10 a.m. and noon is not associated with any concomitant change in the blood sugar.

3. In a series of mice which bore tumours varying from 6 to 20 per cent of their body weights, there was no significant correlation between the blood sugar value and the epidermal mitotic activity.

### REFERENCES.

Bullough, W. S.—(1948) *Proc. Roy. Soc.*, **B**, **135**, 212.—(1949) *J. Exp. Biol.*, **26**, 83. —(1950) *J. Endocrinol.*, **6**, 350.—(1952) *Biol. Rev.*, **27**, 133.

Haslewood, G. A. D., and Strookman, T. A.—(1939) *Biochem. J.*, **33**, 920.

Laws, J. O.—(1952) *J. exp. Biol.*, **29**, 328.

*Idem* and Wright, G. Payling—(1952) *Brit. J. Cancer*, **6**, 236.